# Synergistic Enhancement of Flame Retardancy Behavior of Glass-Fiber Reinforced Polylactide Composites through Using Phosphorus-Based Flame Retardants and Chain Modifiers

**DOI:** 10.3390/polym14235324

**Published:** 2022-12-06

**Authors:** Ceren Yargici Kovanci, Mohammadreza Nofar, Abbas Ghanbari

**Affiliations:** 1 Polymer Science and Technology Program, Institute of Science and Technology, Istanbul Technical University, Maslak, Istanbul 34469, Turkey; 2 Arcelik A.S. Central R&D Department, Polymer & Chemical, Tuzla, Istanbul 34950, Turkey; 3 Sustainable & Green Plastics Laboratory, Metallurgical & Materials Engineering Department, Faculty of Chemical and Metallurgical Engineering, Istanbul Technical University, Maslak, Istanbul 34469, Turkey; 4 National Research Council Canada, 2690 Red Fife Rd., Rosser, MB R0H 1E0, Canada

**Keywords:** polylactide, composites, flame retardant, chain modifier, mechanical properties

## Abstract

Flame retardancy properties of neat PLA can be improved with different phosphorus-based flame retardants (FRs), however, developing flame retardant PLA-based engineering composites with maintained mechanical performance is still a challenge. This study proposes symbiosis approaches to enhance the flame retardancy behavior of polylactide (PLA) composites with 20 wt% short glass fibers (GF). This was first implemented by exploring the effects of various phosphorus-based FRs up to 5 wt% in neat PLA samples. Among the used phosphorus-based FRs, the use of only 3 wt% of diphosphoric acid-based FR (P/N), melamine coated ammonium polyphosphate (APPcoated), and APP with melamine synergist (APP/Mel) resulted in achieving the V0 value in a vertical burning test in the neat PLA samples. In addition to their superior efficiency in improving the flame retardancy of neat PLA, P/N had the least negative effect on the final mechanical performance of PLA samples. When incorporated in PLA composites with 20 wt% GF, however, even with the use of 30 wt% P/N, the V0 value could not be obtained due to the candlewick effect. To resolve this issue, the synergistic effect of P/N and aromatic polycarbodiimide (PCDI) cross-linker or Joncryl epoxy-based chain-extender (CE) on the flame retardancy characteristics of composites was examined. Due to the further chain modification, which also enhances the melt strength of PLA, the dripping of composites in the vertical burning test terminated and the V0 value could be reached when using only 1 wt% PCDI or CE. According to the scanning electron microscopic analysis, the use of noted chain modifiers further homogenized the distribution and refined the particle size of P/N within the PLA matrix. Hence this could synergistically contribute to the enhancements of the fire resistance performance of the PLA composites. Such incorporation of P/N and chain modifiers further leads to the enhancement of the mechanical performance of PLA composites and hence the resultant product can be proposed as a promising durable bioplastic engineering product where fire risk exists.

## 1. Introduction

Global production of fossil-based and non-degradable plastics by many industries continues to increase [1]. This is while a significant amount of greenhouse gasses is also being emitted into the atmosphere during the production process. Such environmental concerns have motivated academia and industry to develop bioplastic alternatives from renewable resources [2,3]. Among bioplastics, due to their similarity to several commodity and engineering fossil-based polymers, polylactide (PLA) and its compounds are considered promising bioplastic alternatives for various applications [4]. In this context, several shortcomings of PLA such as brittleness, low impact strength [5], low service temperature [6], and low thermal stability and flammability still limit its usage in engineering applications [7]. Several studies have disclosed some enhancements in thermal stabilization, flame retardancy, and mechanical performance of PLA and its compounds [8,9]. However, only limited studies address the simultaneous improvement of flame retardancy and mechanical performance of PLA-based fiber-reinforced composites [10].

Different types of flame retardants (FRs) have, so far, been examined to improve the thermal stability and flame resistance of PLA. Similar to those used in conventional polymers, halogenated FRs have been recognized as the most efficient FRs for PLA; however, they are considered hazardous to human health [11]. Hence, their usage tends to be avoided, and halogen-free additives are, nowadays, considered more demanding FRs [12]. Among halogen-free FRs, ammonium polyphosphate (APP) [13], intumescent FRs (IFR) (APP/pentaerythritol/melamine system) [14], ammonium phosphate (AP) [15], triphenyl phosphate (TPP) [16], melamine phosphate (MP) [17], aluminum trihydroxide (ATH) [18], resorcinol bis (diphenyl phosphate) (RDP) [19], aluminum hypophosphite (AHP) [20], hyper-branched polyphosphate ester (HPE) [21], and aluminum phosphinate (AlPi) [22] are the most widely studied FRs for PLA-based plastics. It appears that APP and IFRs are the most effective halogen-free FRs for PLA-based plastics [23,24]. The flame retardancy mechanism of these FRs is through the formation of char onto the condensed phase and the inhibition of flame against the released gas phase [25,26]. In this context, Xue et al. [27] illustrated that the introduction of only 6 wt% APP in PLA is sufficient to obtain the vertical burning value of V0; however, the mechanical performance of the samples was suppressed. They also examined the hybrid effects of ~2 wt% of APP with RDP after which the V0 value was obtained with better mechanical performance. This was because the RDP could also behave as a compatibilizer between APP and PLA which resulted in the finer dispersion of APP particle size.

Due to the high cost of FRs and their destructive effect on the mechanical performance of thermoplastics, it is essential to limit their concentration in the compounds. Hence, the use of synergist agents, which together with FRs may enhance the flame retardancy behavior of PLA, is a promising breakthrough in lowering the FR content and improving the mechanical performance of the compounds [28]. Several synergist agents such as melamine [29], nitrogen compounds [30], lignin [31], nickel oxide [32], hyperbranched charring agents [33], aryl polyphenylphosphonate [34], lignin–silica hybrids (LSH) [35], and the charring agents (CNCA-DA) [36] have been used together with APP to improve the flame retardancy of PLA-based plastics. Some of these synergist additives, such as melamine, encapsulate the APP, and thus the whole encapsulated system behaves as a better-performing FR while the loss in mechanical performance is also minimized [37,38,39]. The clay-based nanoparticles such as sepiolite, modified montmorillonite (O-MMT), and halloysite nanotubes (HNT) have also been incorporated in various studies together with phosphorus FRs as effective synergist agents [40,41]. These nanoparticles decrease the heat release rate and thereby improving thermal stabilization and hindering the melt dripping of the final PLA-based nanocomposites [42,43]. Li et al. [44] demonstrated the synergistic flame retardancy efficiency of the simultaneous use of organically modified montmorillonite (O-MMT) together with IFR in PLA. Results showed that while 20 wt% IFR results in the V2 value in the vertical burning test, the use of 5 wt% O-MMT with only 15 wt% IFR leads to obtaining the V0 value without any dripping. According to the results, it was thought that O-MMT may decrease the heat release of the composite and increase the thermal stabilization thus improving the flame retardancy properties. Since PLA shows heavy dripping during the burning test, anti-dripping agents are also considered as another group of synergist agents to retard the flammability of PLA-based plastics. Zhan et al. [45] extensively studied the effects of various anti-dripping agents together with IFR on PLA flammability. They used O-MMT, zinc borate (ZB), fumed silica (FS), tetraethyl orthosilicate (TEOS), and polytetrafluoroethylene (PTFE) as anti-dripping agents and explored the dripping behavior of PLA-based compounds. They illustrated that the enhanced anti-dripping behavior of samples was indeed correlated with the reduced melt flow index (MFI) values of the compounds at elevated temperatures. In other words, the increase in melt strength and viscosity has a significant contribution to the anti-dripping behavior of the PLA-based systems. Following the same logic, the introduction of chain modifiers that can increase the melt strength may retard dripping and hence improve the flame retardancy behavior of plastics [46,47]. Chen et al. [46] investigated the synergistic effect of zinc oxide (ZnO) together with the Joncryl chain extender (CE) in order to improve the flame retardancy of PLA when APP-based FRs were incorporated. While the use of ZnO with APP could increase the char formation of PLA, the sole addition of CE with APP did not cause further char formation. However, when ZnO was used together with CE, and in the presence of APP, enhancements in limiting oxygen index (LOI) values were reported. They revealed that nano ZnO had a chain-breaking effect during the combustion, which increased the char content and improved flame retardancy. CE addition in the ZnO/APP/PLA system further increased the barrier protection effect as CE provided cross-linking reactions while ZnO provided more reaction sites by chain scission of PLA.

Since glass fiber (GF) reinforced plastics are nowadays considered promising and cost-effective composites for a variety of applications, the enhancement of their flame retardancy is crucial for their usage in engineering applications with high fire risks [48,49]. However, the presence of GF in thermoplastic matrices has an adverse effect on the fire performance behavior due to the candlewick effect [50]. In the combustion process, the polymer melt adsorbs, wets, and flows along the GF surfaces toward the fire. Therefore, capillary action accelerates the heat flow back to the polymers and hence decomposition intensifies and becomes more facilitated. This phenomenon is called the candlewick effect. Hence, it is even more challenging to develop flame-resistant PLA-GF composites than neat PLA-based plastics. Accordingly, in GF-reinforced composites, higher amounts of FRs are needed [51,52]. So far, only a few studies have explored the flame retardancy behavior of PLA-GF composites, which are nowadays considered important alternatives for a series of petroleum-based polymer composites in various engineering applications [13]. Ling et al. [15] showed that 25 wt% AP is required to achieve a V0 flammability value in 20 wt% GF-reinforced PLA/polycarbonate (PC) compounds. According to this study, AP increased the melt viscosity and decelerated the decomposition rate of the PLA/PC blend, while GF also contributed to the flame retardancy of the composite by increasing the melt viscosity of PLA/PC and stabilizing the residual char formed during combustion.

As discussed, many studies have examined the flame retardancy of PLA-based plastics. However, few studies exist on exploring the flame retardancy of GF-reinforced PLA composites. Therefore, in this study, we aimed to develop a flame retardant PLA-based GF-reinforced composite using phosphorus-based flame retardants and chain modifiers to minimize the dripping and improve the flame retardancy of the composite while avoiding suppressions in final mechanical properties. In this context, the effect of six various types of phosphorus-based FRs on the flame retardancy behavior and mechanical properties of neat PLA was first explored. The most effective FR, i.e., diphosphoric acid-based FR (P/N), was then selected to examine the fire and mechanical performances of PLA composites reinforced with 20 wt% short GF. The synergistic effect of P/N FR in the composites modified by two different chain modifiers, aromatic polycarbodiimide (PCDI) cross-linker and Joncryl epoxy-based chain-extender (CE) was explored. Morphology, melt viscosity improvement, as well as the thermal properties of the synergistically modified PLA composites with FR, were also examined in the study.

## 2. Experimental Section

### 2.1. Materials

PLA Purapol L130 grade with MFI of 7 g/10 min (190 °C/2.16 kg) was purchased from Total Corbion, Rayong, Thailand. Short GF with, respectively, average fiber diameter and length of 13 μm and 4.5 mm was supplied from Şişecam Elyaf Sanayi, Istanbul, Turkey. P/N FR, which contains diphosphoric acid with piperazine and nitrogen (ADK STAB^®^ FP-2500S grade), was supplied from Adeka, Tokyo, Japan. APP and melamine-coated APP (APPcoated) were supplied from Eczacıbaşı, Istanbul Turkey. APP with melamine synergist in the mixture form (APP/Mel) JLS-PNP2D grade was supplied from Hangzhou JLS Flame Retardants Chemical Co., Ltd., Hangzhou, China. Melamine phosphate (MP) PreniphorTM EPFR-110DM grade was supplied from Brenntag, Guangdong, China. APP nitrogen mixture (APP/N) Exolit AP 766 grade was provided by Clariant AG, Knapsack, Germany. Aromatic polycarbodiimide (PCDI) was purchased from Lanxess AG, Cologne, Germany. Epoxy chain extender (CE), Joncryl^®^ ADR 4468 was provided by BASF, Germany.

### 2.2. Sample Preparation

All ingredients were dried overnight in a vacuum oven at 80 °C before any process. Formulations were melt compounded in a co-rotating twin-screw extruder (Prism TSE 24 MC, L/D:24). PLA and chain modifiers were fed into the extruder main feeder, while FRs and GF were fed into second and side feeders, respectively. The extruder barrel temperature profile was adjusted at 170–175–180–180–185–190–190 °C (from hopper towards die exit) and the screw speed was fixed at 65 rpm. Neat PLA and different FRs were first compounded and the corresponding formulations are presented in Table 1. GF-reinforced PLA composites with P/N FR with/without PCDI and CE chain modifiers were then processed while GF content was constant in the composition (20 wt%) and the related formulations are also shown in Table 2. Characterization samples were then prepared through a lab-scale Arburg injection molder.

### 2.3. Flame Retardancy Measurements

#### 2.3.1. UL-94 Vertical Burning Test

UL-94 vertical burning tests were conducted in accordance with ASTM D3801 using injection molded specimens with a 130 × 13 × 13 mm^3^ standard size. It was held vertically over the cotton patch, and the flame was applied to the sample for 10 s. The time for the flame to self-extinguish was recorded as t_1_. A second flame (10 s) was applied to the sample, and a second time to self-extinguish was recorded as t_2_. The dripping of samples and burning of the cotton patch caused by the dripping during the burning test were also recorded. American National Standard UL-94 was used to define classifications.

#### 2.3.2. LOI Test

LOI measurements were carried out on the PLA composite samples using FTT (Fire Testing Technology, East Grinstead, UK) oxygen index machine in accordance with ISO 4589-2. The dimension of the injection molded composites was 80 × 10 × 4 mm^3^. In this test, the specimen is placed vertically in the transparent chimney where a mixture of oxygen and nitrogen flows upwards through it. Then an ignition source contacts the top end of the specimen and is withdrawn. If ignition occurs, the oxygen concentration in the column is reduced for the next specimen. If the specimen does not ignite, the oxygen concentration is increased for the next specimen. Minimum oxygen concentration is determined by testing a series of specimens.

### 2.4. Mechanical Performance

#### 2.4.1. Tensile Test

Zwick Z 020 with a 25 kN load cell model drawing device was used for tensile tests at room temperature, and a 5 mm/min drawing speed was applied. Dimensions of the injection molded dog bone samples were 3 mm thickness, 4 mm width, and 20 mm gage length. Five specimens from each case were tested, and the average value was used in the results. Tensile properties of the samples were performed in accordance with 527-1.

#### 2.4.2. Flexural Test

The Instron 5900 R with a 25 kN load cell was also used to conduct a three-point bending test according to ISO 178. Dimensions of the injection molded samples were 4 × 80 × 10 mm^3^. Tests were conducted at a bending speed of 3 mm/min. Five specimens from each case were tested, and the average values are reported.

#### 2.4.3. Impact Test

The Izod notched impact strength was measured with a Zwick Roell HIT 5.5P device in accordance with ISO 180/A1. Dimensions of the samples were 4 × 80 × 10 mm^3^. Ten specimens were tested for each sample, and the average values are reported.

### 2.5. Melt Flow Index (MFI)

MFI of neat PLA and PLA compounds were performed using Instron ceast MF30 tester at 190 °C and 2.16 kg according to ISO 1133.

### 2.6. Dynamic Mechanical Analysis (DMA)

Dynamic mechanical analysis (DMA) of the injection molded samples was performed by using a TA Instruments, DMA Q 800 dynamic mechanical analyzer under dry air in tension mode. The temperature sweep experiments were conducted at the temperature range from 30 and 130 °C and the heating rate of 2 °C/min at a constant frequency of 1 Hz.

### 2.7. Scanning Electron Microscopy (SEM)

The morphology studies of PLA compounds were analyzed using an SEM (ZEISS, model: SUPRA 55VP) with an acceleration voltage of 10 kV. Dispersion and particle size of FR additives in PLA matrix were observed from fractured surfaces of samples.

### 2.8. Thermogravimetric Analysis (TGA)

TGA was performed by using a TA instrument TGA Q500. The test samples were heated from room temperature to 900 °C with a heating rate of 10 °C/min. Each test was conducted under a nitrogen atmosphere.

## 3. Results and Discussion

### 3.1. Performance of Neat PLA with Various FRs

#### 3.1.1. Flame Retardancy Behavior

The flame retardancy of PLA and different phosphorus-based FRs (i.e., APP, APP/N, APPcoated, APP/Mel, MP, and P/N) were evaluated through the UL-94 vertical burning test. Table 3 shows the vertical burning test results of the neat PLA compounds with 3 or 5 wt% FR contents. During the vertical burning test, neat PLA burned continuously without char formation and revealed dense dripping, which ignited the cotton ball and caused the test to fail. In PLA/5 wt% FR compounds, except PLA-MP, all cases reached the vertical burning test value of V0. When the FR amount decreased to 3 wt%, only PLA compounds with P/N, APPcoated, and APP/Mel formulations reached the V0 burning test value while producing a dense char layer on the combustion surface. The results show that the use of melamine in APP FR increases the flame retardancy performance of PLA. A similar significant result has been reported by Sun et al. [53]. It was found that APP and melamine have synergy in the condensed phase and they showed better flame retardancy performance when they were used together. They observed significant HRR reduction with 5 wt% Mel and 25 wt% APP mixture used in the PLA compound. Similar synergy was observed in this study in the APP/Mel and APP/Coated formulation.

#### 3.1.2. Mechanical Properties

The effect of various FR additives (i.e., APP, APP/N, APPcoated, APP/Mel, MP, and P/N) at a loading of 20 wt% on mechanical performance (i.e., tensile, flexural, and impact tests) of PLA compounds was examined. As incorporating 20 wt% FR in PLA/GF composites was inevitable, such high FR content had to be introduced to the neat samples for evaluating mechanical properties. The corresponding results are presented in Table 4. Compared to the neat PLA, the addition of 20 wt% FR decreased the tensile strength and increased the tensile modulus. A slight increase was observed in the Izod notched impact strength of PLA-APP and PLA-P/N formulations, while all other FRs had an adverse effect on Izod notched impact results. In all, considering the tensile, flexural, and impact test results, the incorporation of P/N FR in PLA showed the least destructive effect while the V0 value is already reached with the use of only 3 wt% P/N. Therefore, the performance investigation of the PLA/GF composite was conducted by incorporating only P/N FR due to its more effective role in enhancing flame retardancy behavior with a minimized destructive effect on the mechanical performance of the PLA samples. Of note, due to their brittle nature, we could not measure the tensile and impact values of the injection molded samples containing APP/Mel FR. This is while the flexural strength of PLA-APP/Mel samples dramatically reduced.

### 3.2. Performance of PLA/GF Composites with P/N FR and with/without Chain Modifiers

#### 3.2.1. MFI Results

**Figure 1:**
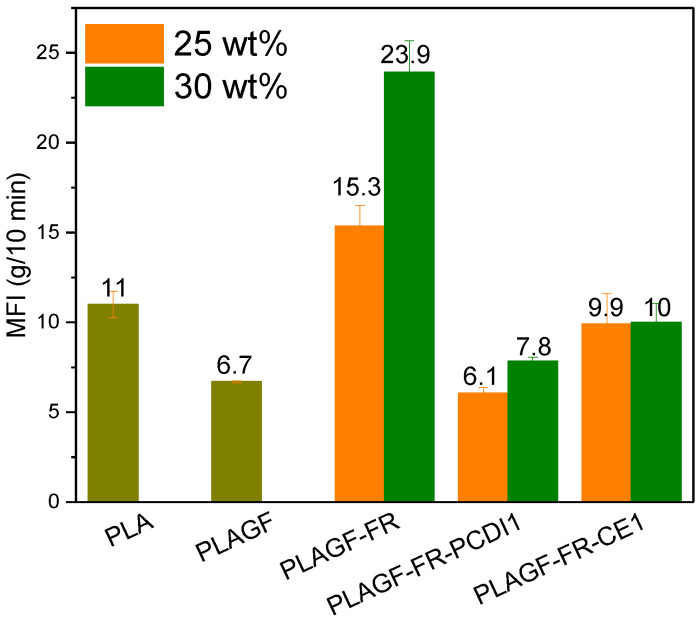
MFI results of PLA and PLA composites with and without FR and chain modifiers at 190 °C and 2.16 kg.

MFI results of the neat PLA and PLA composites with and without FR and PCDI or CE chain modifiers are shown in Figure 1. While neat PLA revealed an MFI value of 11 g/10 min, the addition of 20 wt% GF reduced this value to around 6.7 g/10 min. With the addition of 25 or 30 wt% of P/N in PLA/GF composites, the MFI values dramatically increased up to around 15.3 and 23.9 g/10 min, respectively. The small molecules of FR could plasticize the PLA molecules and hence decrease the viscosity of PLA composites [45]. This indicates while the addition of P/N FR could improve the flame retardancy behavior of the composites, it could also increase the flowability of the material. In contrast, with the incorporation of only 1 wt% of chain modifiers (i.e., PCDI or CE), the MFI of the PLA/GF/FR systems could noticeably decrease. As depicted in Figure 1, the use of 1 wt% PCDI or CE decreased the MFI values to around 6.1 and 9.9 g/10 min, respectively, when 24 wt% FR P/N was used. Such values were 7.8 and 10 g/10 min, respectively, in samples with 29 wt% FR P/N. Such a decrease in MFI is due to the chain extension and/or branching of the PLA molecules through the reacting end groups of these two modifiers [54]. Therefore, these reactions lead to a decrease in the flowability of polymer chains and result in an increase in polymer viscosity. Hence when exposed to fire, and upon melting, the material dripping could be hindered with such viscosity increase.

**Figure 2:**
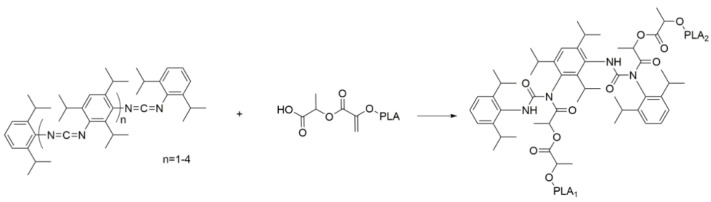
Possible reaction between PLA and the PCDI [55].

**Figure 3:**
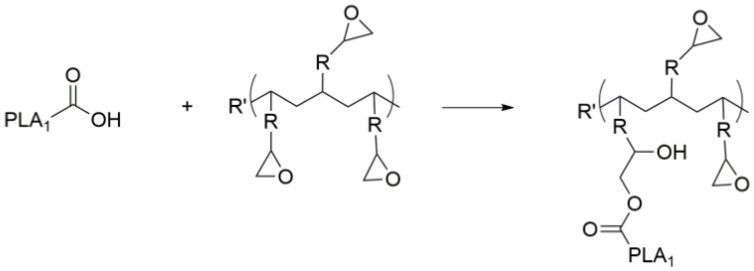
The possible reaction between PLA and the epoxy chain extender [47].

The possible reaction mechanism between chain modifiers and PLA is shown in Figure 2 and Figure 3, respectively. Both PCDI and CE chain modifiers can react with hydroxyl and carboxyl groups of PLA and increase the molecular weight of the PLA [55]. While PCDI can react only through the linear direction, CE can perform multifunctional reactions due to its multifunctional structure. Thus, it increases the viscosity of PLA more than the PCDI additive [47]. Therefore, the MFI results of CE PLA composites are lower than PCDI PLA composites.

#### 3.2.2. Flame Retardancy

The flame retardancy properties of neat PLA, PLA reinforced with 20 wt% GF, and the noted composites with P/N FR and with/without chain modifiers were examined through UL-94 vertical burning and LOI tests, and the results are shown in Table 5. During the burning test of PLA-GF composite without FR, the samples did not self-extinguish, but dropped heavily onto the cotton ball and caused its ignition without char formation. On the other hand, while all P/N FR-containing composites formed foamed char, the cases with P/N FR < 25 wt% could not be self-extinguished. This is while in the compounds containing 25 wt% P/N FR, the samples were self-extinguished after more than 30 s of burning (burning time between 35 s to 100 s), although eventually, the cotton ball was ignited by the droplet, and the test failed. In composites with 30 wt% P/N FR, the composites were self-extinguished within 30 s which resulted in achieving a V1 burning test value.

With the addition of only 1 wt% chain modifiers, it was interestingly illustrated that all these behaviors are remarkably influenced. The addition of 1 wt% PCDI increased the flame retardancy of PLA-GF composite with 29 and even 24 wt% of P/N. In the UL-94 vertical burning test, PCDI formulations did not ignite at t_1_ and t_2_, and dripping stopped completely even at 24 wt% FR loading. Moreover, the addition of 1 wt% CE additive in the PLA-GF formulation provided self-extinguished and anti-dripped PLA composition both when FR contents of 24 and 29 wt% were incorporated. Hence, in all chain-modified composites with either 24 or 29 wt% of P/N FR, V0 values are reached in vertical burning tests. The chain modification of PLA molecules through PCDI and CE causes a serious increase in viscosity and hinders molecular mobility. Hence, when exposed to fire, the hindered molecules’ mobility and significantly increased chain entanglements decelerate the dripping when the samples are exposed to fire, for instance, in the vertical burning test [44]. This is consistent with the results obtained by Zhan et al. [45] who found a relation between melt dripping behavior during a combustion test with MFI values of PLA IFR composites. They revealed that the flowability properties of the polymer composite at high temperatures have a strong effect on the anti-dripping characteristic of the composite.

**Figure 4:**
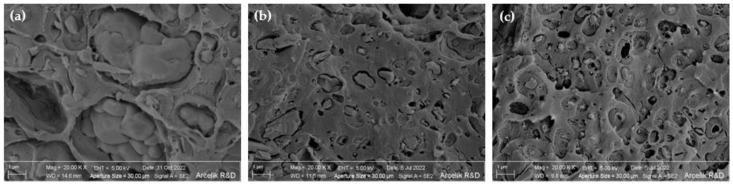
SEM images of impact fracture surfaces: (**a**) PA-GF-25FR; (**b**) PLA-GF-24FR-1PCDI; (**c**) PLA-GF-24FR-CE (the scale bar is 1 μm).

Furthermore, the SEM images (Figure 4) reveal that the increased viscosity of the PLA through the chain extension and/or branching leads to the more uniform dispersion of the P/N FR within the PLA matrix with finer particle size. As shown, some P/N FR agglomerates could be observed in composite samples without the chain modifiers. The addition of 1 wt% of the chain modifiers, specifically CE, caused the formation of finer particles with more homogenous dispersion of P/N FR in the PLA matrix. The particle size of the P/N FR decreased from around 1.6 μm to about 0.9 μm and 0.3 μm when using 1 wt% PCDI and CE, respectively. Therefore, finer P/N FR with a more homogenous distribution contributes more significantly to the improvement of the flame retardancy behavior of the samples [56]. Such chain modifiers’ behavior (i.e., their anti-dripping behavior and improver of FR dispersion) together with the presence of finely dispersed P/N FR could hence, significantly create a synergy to enhance the flame retardancy behavior of PLA/GF composites.

The LOI test results of PLA composites are also presented in Table 5. When FR was introduced into PLA/GF formulations, the LOI values increased from 22% in PLA/GF to 43.5%, 44%, and 49.7%, in PLA/GF with 20, 25, and 30 wt% P/N FR, respectively. The use of PCDI and CE chain modifiers in PLA/GF with 24 and 29 wt% P/N FR, however, did not reveal a significant improvement in the LOI values, and similar values to those unmodified samples were obtained.

**Figure 5:**
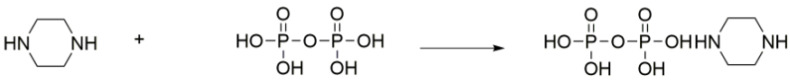
Piperazine pyrophosphate [59].

**Figure 6:**
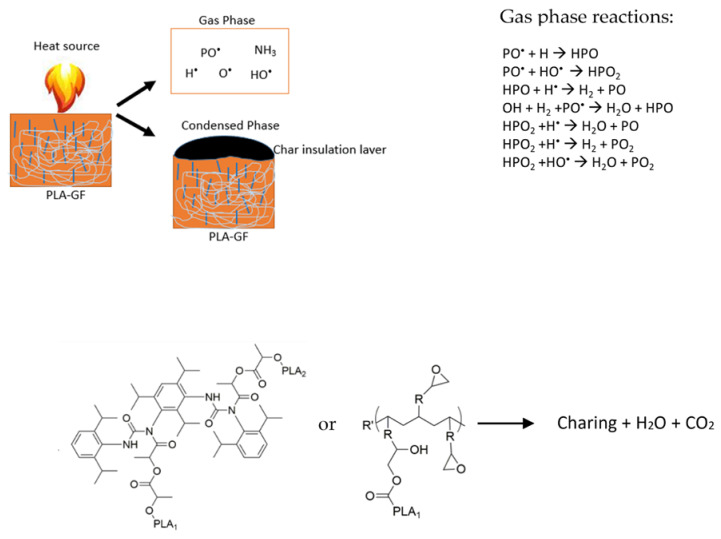
Possible flame retardant mechanism of PLA GF composites.

The flame retardant structure of P/N FR in the PLA matrix is illustrated in Figure 5 and a possible flame retardant mechanism is illustrated in Figure 6. During the combustion process of neat PLA, it decomposes to generate lots of small molecule compounds like alkanes, aldehydes, lipids, and cyclic oligomers [57]. In the case of P/N FR incorporation in the PLA composite, flame retardancy properties significantly improved. As illustrated in Figure 6, the flame retardancy mechanism of P/N acts in the condensed phase and also the gas phase. The P/N FR decomposed and released PO⠁, P⠁ and NH_3_ in the gas phase. These radicals capture the gaseous active radicals (O⠁, H⠁, and OH⠁), which are produced during PLA pyrolysis, thus, hindering the combustion process in the gas phase. At the same time, inert gases like NH_3_ diluted the concentration of the flammable volatiles in the gas phase. Furthermore, P/N decomposition formed a dense char layer in the condensed phase which acts as an insulation layer between the combustion process and the PLA surface [58]. Chain-extended PLA composites particularly increase chain entanglements and melt strength, thus hindering molecular mobility which especially contributes to preventing the dripping of polymer chain when the heat source is applied. Furthermore, the aromatic groups of PCDI and epoxy groups of CE may also contribute to the flame retardancy of the PLA composite.

#### 3.2.3. Mechanical Properties

Table 6 reports the tensile and flexural strength and modulus and Izod notched impact strength results of the composites. The addition of 20 wt% GF significantly increased the strength and modulus values of PLA. The tensile modulus and impact strength of the neat composite increased around 80% and 40%, respectively, with respect to those of neat PLA. When P/N FR was also incorporated in the composites, the tensile strength and Izod strength were decreased by 17% and 37% compared to those of the neat composite, respectively, and the tensile modulus was increased by 37% in the case when 24 wt% FR was used. The reason is, since FR performed like a filler in the composite, it can also enhance the material rigidity. More importantly, the incorporation of PCDI and CE additives increased the tensile strength and Izod notched impact strength values significantly. These results are related to the increased molecular weight and formation of a long-chain branching structure [60]. The chain’s extended or branched structure increases the polymer chains’ entanglement thus this hampers the movement of polymer chains [61]. As seen, the use of PCDI or CE chain modifiers reduces the adverse effect of P/N FR on the mechanical properties of the composites.

#### 3.2.4. DMA Results

**Figure 7:**
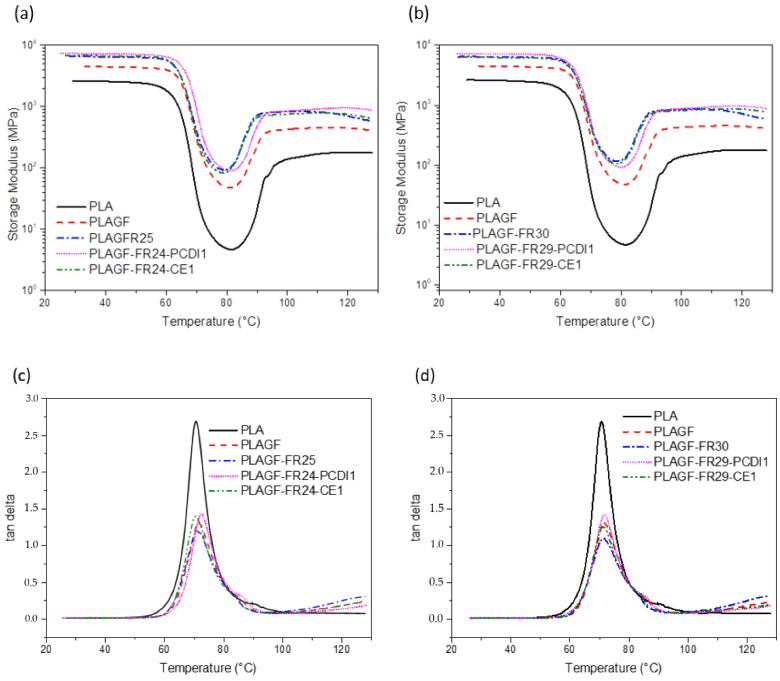
Storage modulus comparison of PLA composites with (**a**) 25 wt% and (**b**) 30 wt% FR; and tan δ graphs with (**c**) 25 wt% and (**d**) 30 wt% FR.

Figure 7 shows the dynamic mechanical properties of PLA/GF composites with 25 and 30 wt% P/N FR with and without 1 wt% PCDI or CE. As seen, the addition of GF to PLA significantly increased the modulus of PLA below its glass transition temperature. The P/N FR addition even more significantly enhanced the modulus of PLA/GF composites. The P/N FR addition even more significantly enhanced the modulus of PLA/GF composites. This result demonstrated that P/N FR increased the stiffness of the composite since P/N particles hinder the mobility of polymer chains [18]. Although with the addition of 1 wt% CE into the PLA/GF/FR systems no significant changes were observed, in the composite systems with 1 wt% PCDI, a noticeable modulus improvement was monitored. According to Figure 7c,d, P/N incorporation into PLA-GF composite led to a decrease in tan δ magnitude, which is associated with the damping properties of the system. The use of 1 wt% PCDI or CE in the composite resulted in a higher tan delta in PLA-GF-FR systems. There is not a significant difference between the tan delta temperatures of the samples, which indicates no explicit change in the glass transition temperature of PLA composites.

#### 3.2.5. TGA Results

**Figure 8:**
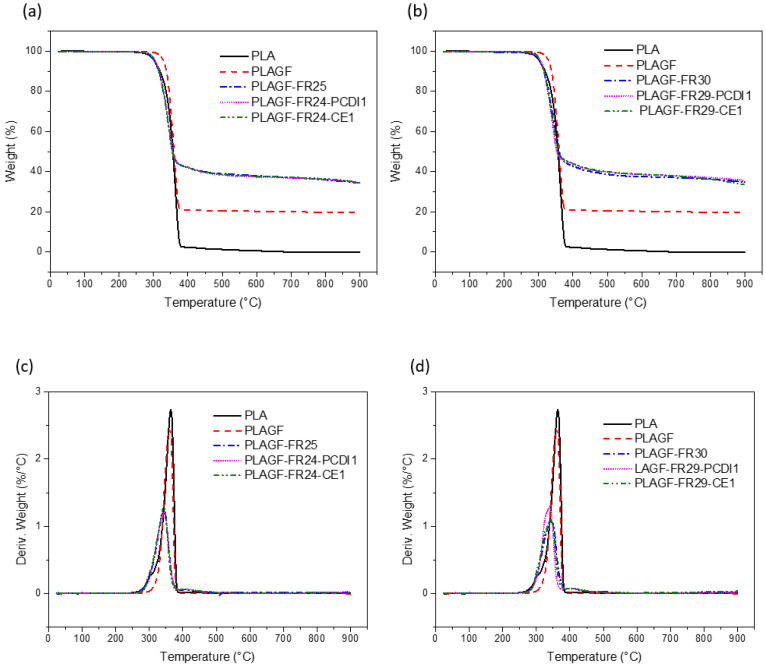
TG curves of the neat PLA and PLA-GF composites (**a**) comparison with 25 wt% FR content (**b**) comparison with 30 wt% FR content; and DTG graphs with (**c**) 25 wt% and (**d**) 30 wt% FR.

The thermal stability of the samples was investigated by TGA and the results are shown in Figure 8 and Table 7. The T_onset_ temperature represents the temperature of 5% mass loss. The addition of 20 wt% GF into the neat PLA increased the T_onset_ from 281 °C to 318 °C. However, usage of P/N decreased the T_onset_ of PLA composites. T_onset_ of PLA decreased to 285 °C and to 288 °C in composites with, respectively, 25 and 30 wt% FR indicating an insignificant effect of FR content on T_onset_. The use of PCDI and CE additives in PLA-GF composites improved T_onset_ temperature by about 5 °C. These additives improved the thermal stabilities of the composite due to the increased molecular entanglement and branching degree [62,63]. When 25 wt% P/N was used in the composites, the char residue at 600 °C was around 37% which includes 20 wt% of the residual GF. The use of PCDI and CE additives, however, did not have a significant influence on the residual char content.

## 4. Conclusions

The performance of five different phosphorus-based FRs on neat PLA was analyzed in terms of flame retardancy and mechanical properties. Among all, APP/Mel, APPcoated, and P/N FRs reached the V0 value in the vertical burning test with the usage of only 3 wt% FR addition in PLA. Considering the durability, it was observed that the adverse effect of the FR additive on the mechanical properties was the least in the P/N additive among all FR additives. P/N additives were further examined in the 20 wt% GF-reinforced PLA composites. A higher amount of P/N FR was needed to reach high flame retardancy in GF-reinforced PLA composites since GF obstructed the polymer flame resistance. Even when the P/N amount increased up to 25 wt%, samples failed the test due to dripping and burning for more than 30 s in the vertical burning test. However, CE or PCDI chain modifiers showed significant improvement and led to achieving the V0 flammability rating in PLA composites containing 24 wt% P/N and 20 wt% GF, without any dripping in the vertical burning test. According to the SEM images, chain modifiers in PLA-GF composites prevented agglomerations and provided fine and homogenous distribution of FR additives. This improvement in morphology contributed to the flame retardancy performance of FR in PLA composites. Chain modifiers performed end-group reactions in PLA polymer chains and decreased MFI values from 15.3 to 6.1 and 9.9 g/10 min with 1 wt% PCDI and CE addition, respectively, for 24 wt% FR PLA-GF composites. This enhancement in viscosity and melt strength also contributed to the anti-dripping behavior of the chain-modified PLA composites.

20% GF reinforcement provided superior mechanical properties to PLA. Even though FR usage caused a decrease in impact strength and tensile strength of the PLA-GF composites, chain modifiers minimized adverse effects of FR in tensile, flexural strengths and Izod notched strength properties. Similarly, P/N additives had a negative effect on the PLA composite’s decomposition temperature, it decreased T_onset_ values by about 33 °C according to the TGA test results. Chain modifiers elevated T_onset_ temperatures by about 5 °C as a result of increased molecular entanglement and branching degree in the PLA composites.

In conclusion, with optimum FR and chain extender additive, superior mechanical and flammability performance were provided in the PLA-GF composite. This developed formulation is a great bio-based plastic alternative for the durable appliances industry, especially parts like electronic card holders, insulation parts, barrier plastics, etc.

## Figures and Tables

**Table 1:** PLA with different FRs and their corresponding compositions.

Samples	PLA (wt%)	MP (wt%)	APP/N (wt%)	P/N (wt%)	APP/Mel (wt%)	APP (wt%)	APPcoated (wt%)
PLA	100						
PLA-MP5	95	5					
PLA-APP/N5	95		5				
PLA-P/N5	95			5			
PLA-APP/Mel5	95				5		
PLA-APP5	95					5	
PLA-APPCoated5	95						5
PLA-MP3	97	3					
PLA-APP/N3	97		3				
PLA-P/N3	97			3			
PLA-APP/Mel3	97				3		
PLA-APP3	97					3	
PLA-APPCoated3	97						3

**Table 2:** PLA and PLA composite with PN FR and with/without chain modifiers.

Samples	PLA (wt%)	GF (wt%)	P/N (wt%)	PCDI (wt%)	CE (wt%)
PLA	100				
PLAGF	80	20			
PLAGF-FR15	65	20	15		
PLAGF-FR20	60	20	20		
PLAGF-FR25	55	20	25		
PLAGF-FR30	50	20	30		
PLAGF-FR24-PCD1	55	20	24	1	
PLAGF-FR29-PCD1	50	20	29	1	
PLAGF-FR24-CE1	55	20	24		1
PLAGF-FR29-CE1	50	20	29		1

**Table 3:** UL-94 vertical burning test results of PLA with different FRs.

Samples	Dripping	Ignition of Cotton	UL-94 Rating
PLA	Yes	Yes	V2
PLA-MP5	Yes	Yes	V2
PLA-APP/N5	Yes	No	V0
PLA-P/N5	Yes	No	V0
PLA-APP/Mel5	Yes	No	V0
PLA-APP5	Yes	No	V0
PLA-APPCoated5	Yes	No	V0
PLA-MP3	Yes	Yes	V2
PLA-APP/N3	Yes	Yes	V2
PLA-P/N3	Yes	No	V0
PLA-APP/Mel3	Yes	No	V0
PLA-APP3	Yes	Yes	V2
PLA-APPCoated3	Yes	No	V0

**Table 4:** Mechanical test results of PLA with different FR formulas.

Samples	Tensile Strength (MPa)	Tensile Modulus (MPa)	Flexural Strength (MPa)	Flexural Modulus (MPa)	Izod Notched Impact Strength (kJ/m^2^)
PLA	69 (±1.2)	3552 (±150)	102.45 (±1.35)	3025 (±20)	3.08 (±0.3)
PLA-MP20	55.96 (±1.42)	3961 (±218)	94.3 (±1.42)	4070 (±12)	2.77 (±0.49)
PLA-APP/N20	47,46 (±2.17)	3578 (±147)	59.1 (±1.16)	3710 (±91)	2.07 (±0.38)
PLA-P/N20	53.87 (±1.77)	4292 (±160)	92.2 (±2.05)	4080 (±75)	3.35 (±0.35)
PLA-APP/Mel20	NR	NR	30.4 (±3.22)	3810 (±136)	NR
PLA-APP20	41.32 (±1.58)	3971 (±158)	74.5 (±1.86)	3900 (±120)	3.32 (±0.14)
PLA-APPCoated20	47.96 (±1.09)	4326 (±90)	69.4 (±0.8)	4070 (±63)	2.9 (±0.21)

**Table 5:** UL-94 vertical burning and LOI results of PLA and PLA composites.

Samples	t_1_ (s)	t_2_ (s)	Dripping	Ignition of Cotton	UL-94 Rating	LOI (%)
PLA	Burn-to-clamp	-	Yes	Yes	NR	20.2
PLAGF	Burn-to-clamp	-	Yes	Yes	NR	22
PLAGF-FR15	Burn-to-clamp	-	Yes	Yes	NR	-
PLAGF-FR20	Burn-to-clamp	-	Yes	Yes	NR	43.5
PLAGF-FR25	0	>30	Yes	Yes	NR	44
PLAGF-FR30	0	30	Yes	No	V1	49.7
PLAGF-FR24-PCD1	0	0	No	No	V0	46.4
PLAGF-FR29-PCD1	0	0	No	No	V0	50.6
PLAGF-FR24-CE1	0	0	No	No	V0	45.3
PLAGF-FR29-CE1	0	0	No	No	V0	49.9

**Table 6:** Mechanical properties of PLA and PLA composites.

Samples	Tensile Strength (MPa)	Tensile Modulus (MPa)	Flexural Strength (MPa)	Flexural Modulus (MPa)	Izod Notched Impact Strength (kJ/m^2^)
PLA	69 (±0.88)	3551 (±190)	102.5 (±3.8)	3025 (±310)	3.08 (±0.16)
PLAGF	59.15 (±0.64)	6420 (±145)	93.7 (±2.8)	6980 (±215)	4.4 (±0.14)
PLAGF-FR25	49.88 (±0.74)	8197 (±206)	69.4 (±6.49)	9700 (±305)	2.78 (±0.11)
PLAGF-FR24 PCDI1	51.55 (±0.94)	7709 (±181)	77.8 (±1.8)	10,100 (±269)	2.93 (±0.11)
PLAGF-FR24-CE1	54.07 (±0.59)	7578 (±113)	79 (±1.28)	9700 (±208)	2.98 (±0.13)
PLAGF-FR30	46.6 (±0.74)	8471 (±166)	53.3 (±4,86)	10,000 (±335)	2.48 (±0.08)
PLAGF-FR29-PCDI1	52.61 (±0.85)	8046 (± 84)	70.4 (±2.45)	10,300 (±158)	2.7 (±0.06)
PLAGF-FR29-CE1	58.68 (±0.47)	8332 (±129)	76.3 (±2.57)	9540 (±346)	2.72 (±0.09)

**Table 7:** TGA data of the PLA and PLA-composites.

	T_onset_ (°C)	T_max_ (°C)	Residue at 600 °C (%)
PLA	281.1	363.9	0.4
PLAGF	318.4	360.8	20.3
PLAGF-FR20	294.4	350.3	34.6
PLAGF-FR25	285.5	342.7	38.0
PLAGF-FR24-PCDI1	290.1	344.1	37.4
PLAGF-FR24-CE1	290.9	342.9	37.7
PLAGF-FR30	288.4	345.2	37.5
PLAGF-FR29-PCDI1	292.1	338.7	38.6
PLAGF-FR29-CE1	292.4	343.4	38.8

## Data Availability

Not applicable.
